# A prospective study on direct out-of-pocket expenses of hospitalized patients with acute exacerbation of chronic obstructive pulmonary disease in a Philippine tertiary care center

**DOI:** 10.1186/s12890-024-03011-y

**Published:** 2024-04-17

**Authors:** Blake Warren Ang, Lenora Fernandez

**Affiliations:** Division of Pulmonary Medicine, University of the Philippines? Philippine General Hospital, Manila, Philippines

**Keywords:** COPD, Exacerbations, Cost of hospitalization

## Abstract

Chronic obstructive pulmonary disease (COPD) is a frequent cause of morbidity and mortality in the Philippines and majority of the economic burden lies in hospitalizations during an exacerbation. Despite coverage of hospitalization cost with the national health insurance system (Phil-Health) for COPD exacerbations, patients often pay out-of-pocket. This study aimed to determine the demographic characteristics of COPD admissions at a Philippine tertiary care center, Philippine General Hospital, and assess mean cost of hospitalization, and identify predictors of prolonged hospitalization and cost > 20,000 Philippine pesos (Php). A prospective cross-sectional study was conducted for 6 months by chart review. Patients were categorized as charity service patients, that is, with no charged professional fees and free medications and private service patients who pay for their health care services. A total of 43 COPD admissions were included. The average daily cost of hospitalization (at peso-dollar rate of 56) for service patients was at $ 75.89 compared to private service patients at $ 285.71. Demographic characteristics and type of accommodation were not significant predictors of prolonged hospital stay nor hospitalization cost of ≥ $ 357. Accommodation cost and professional fees accounted for majority or 61.6% of the overall cost for private patients, while medications and diagnostic tests were the major or 76.01% contributor to the overall cost for charity patients. Despite existence of Phil-health, in-patient coverage for COPD remain insufficient. Measures for maximizing COPD control in the out-patient setting could potentially reduce total cost for this disease.

## Introduction

Chronic obstructive pulmonary disease (COPD) is an increasing cause of morbidity and mortality in the Philippines. Estimates of the disease burden project COPD to be the 3rd leading cause of mortality worldwide [1–3]]. Majority of the economic burden lies in the cost of hospitalizations during a COPD exacerbation [4]. With the new national health insurance structure, diseases are given specific case rates, and allows for faster disbursement of claims [5]. 

According to WHO, there about 65 million people suffering from moderate to severe chronic obstructive pulmonary disease (COPD) out of the 328 million estimated globally [1–2]. Projected estimates report COPD to become the 3rd leading cause of death worldwide by 2030 with the majority of the reported deaths in COPD unfortunately occurring among low to middle-income countries [2–3]. Based on local studies of the disease in the Philippines in 2012, the COPD prevalence is reported to be at least 14%, whereas in other ASEAN countries, the overall estimated COPD prevalence was 6.2%, with 19.1% of subjects having severe COPD [6–7]. Smoking is one of the implicated risk factors for disease development, and smoking in the Philippines is noted to be higher at 28.3% than its ASEAN counterparts. This translates to at least 17 million of smoking adult Filipinos [8]. 

COPD presents with chronic cough, sputum production and dyspnea on exertion among patients with moderate to severe disease. The disease is characterized by airflow limitation that is not fully reversible [9]. Although multiple therapies are already available, the disease remain underdiagnosed and undertreated especially among primary care physicians [10–11]. Undertreated and uncontrolled COPD is marked by frequent exacerbations defined as an acute event of worsening respiratory symptoms requiring medications [8]. Markers for increased risk of exacerbations based on studies include old age, chronic mucus hypersecretion, and decreased Forced expiratory volume in one second (FEV1). Moreover, COPD is progressive and end-stage disease is characterized by dyspnea even at rest with eventual respiratory failure [12]. 

Important factors that influence the health care costs of COPD in general include disease severity, frequency of exacerbations, and the presence of comorbidities [6, 13]. The predominant economic burden lies in the hospitalization costs for COPD exacerbations which is linked to higher mortality rates [6]. In the US, exacerbations account for $18 billion in direct costs annually [14]. This high cost of therapy appears universal based on several studies conducted in Asia [15–17]. Treatment must therefore focus on the control of symptoms and prevention of future exacerbations– with emphasis on the use of maintenance inhaler therapies. This poses a problem among lower middle-income countries like the Philippines, which utilize health care resources to respond mainly to acute illnesses, and are less adept at addressing chronic conditions such as COPD [18]. 

National health coverage initially came into law through the Philippine Medical Care Act of 1969 which was signed by President Ferdinand E. Marcos, eventually implemented in 1971. This act provided total coverage of medical services according to the needs of the patients. During the term of President Fidel V. Ramos, the development of House Bill 14,225 and Senate Bill 01738 enabled the development of National Health Insurance Act of 1995 (RA 7875). This created the Philippine Health Insurance Corporation (PhilHealth) [19]. As of 2011, the case rate system has been introduced under the administration of President Benigno Aquino III. This payment mechanism set pre-determined coverage for select medical conditions and surgical procedures [5]. This significantly reduced the turnaround time or processing of claims since the amount of coverage is already identified. The new case rate system however may cause out-of-pocket expenses, with the present coverage for chronic obstructive pulmonary disease set at only $ 217.86 [19]. 

This study aims to identify the amount of out-of-pocket expenses by patients admitted for COPD exacerbation at the Philippine General Hospital and predictors for prolonged stay and hospitalization costs. This will determine the economic burden of patients who are admitted with COPD in Philippine General Hospital as well as provide financial estimates on the direct costs of treatment for COPD exacerbations over the case rate provided by Phil-Health insurance.

## Objective of the study

The objectives of this study were to determine the out-of-pocket expenses of adult patients admitted for COPD in acute exacerbation using direct costing. The general demographics of the confined COPD patients were gathered, the mean cost of hospitalization due to COPD exacerbation among private and charity patients were compared, the relationship between baseline demographic characteristics and costs was evaluated and the predictors of hospitalization cost and prolonged hospital stay of COPD in exacerbation were determined.

## Scope and limitation of the study

As a pilot study, it was conducted only at the Philippine General Hospital from June 2019 to November 2019 and only those cases from the said period were included in the sample. The study may not be conclusive because of the small sample but it will lead to the performance of a national-scale research project by including other hospitals in the country. The data extraction was done by the research coordinator who collects data daily. The cases are admitted under the internal medicine department and a census review is done to identify the COPD cases admitted.

## Background information and brief literature review

Chronic Obstructive Pulmonary Disease (COPD), the seventh leading cause of death in the Philippines, has a destructive impact on patients’ function and quality of life, and increases the risk of developing heart disease, lung cancer, and a variety of other conditions. One of 10 Filipino adults aged 40 and up may have COPD and a mere 2% of them will be clinically diagnosed [23]. 

In the case of highly prevalent chronic diseases, it is essential to quantify the social and financial magnitude of the disease in all spheres (direct and indirect costs, health and non-medical costs, labor losses and intangible costs). This will improve the ability to design, prioritize, and apply preventive and health promotion measures, investments in public health or social policies and protocols and care guidelines in the most equitable and efficient way possible. Furthermore, this will also allow constant updating to prevent current practices from becoming obsolete, inefficient, and costly because they are not adapted to the reality of the disease [32]. 

COPD hugely affects productivity in the workforce. Income is lost for the inflicted. For a worker who misses 7 days’ work while being treated or recovering, that is at least $ 64 down the drain. Most often, an employed family member also skips work to watch over the patient, incurring the loss of wages twice. The devastating impact of COPD doesn’t stop there as sometimes, children quit school and/or work to provide added source of income. Worst still, with our country’s life expectancy of 67 years, a COPD patient who dies at 50 loses 17 potential years where he could have earned around PHP 2.8 million (based on the minimum daily wage of $ 9.14 for NCR and a peso dollar rate of 56) [24]. 

Patients are charged with room and professional fees upon discharge from private service accommodation. Charity service accommodations are non-airconditioned wards which are generally shared with other admitted patients by specialty (Medicine ward, Surgery ward, Orthopedics ward, Gynecology ward, etc.) and are stationed by resident physicians and fellows in training. Specialist attending physicians serve charity wards by conducting regular teaching rounds with the trainee physicians and provide guidance in the management. Charity patients do not have the burden of cost for accommodation and medical services. Hospital accomodation means accommodation at any hospital or duly registered medical facility, including all meals and refreshments. Adult patients ≥ 19 years old with the main diagnosis of COPD and/or PhilHealth enrollment claims using the ICD-10-CM Diagnosis Code J44.9 were included. Patients who were ≤ 18 years old, not having COPD or ICD code J44.9 as being one of the primary assessment on admission and those who developed COPD exacerbation only during the hospital stay were excluded from the study.

The Phil-health case rate is the fixed rate or amount that PhilHealth will reimburse for a specific illness/ case, which shall cover for the fees of health care professionals, and all facility charges including, but not limited to, room and board, diagnostics and laboratories, drugs, medicines, and supplies, operating room fees and procedures, regardless of member category, that are admitted in accredited health care institutions. In the case of COPD, the total case rate is equivalent to $ 435.71 at peso-dollar rate of 56. This includes $ 217.86 for the case rate, $ 65.36 for professional fee and $150.89 for healthcare institution fee.

Diagnostic and medication expenses for co-morbidities apart from COPD were included as direct costs of the hospitalization. Costing for charity patients under the No Balance Billing system was derived similar to other charity patients, and the same case rate was implemented. No balance billing system is simply means hospitals or medical providers cannot charge a higher price than the amount the insurance agreed to pay. External funding of charity patients from third party financial sources inside PGH were considered as excess costs (hypothetical) for this study based on the formula expressed above. The concept of prolonged stay comes from an empirical observation that as patients stay longer in the hospital [25]. Direct cost is a cost of a resource or activity that is acquired for or used by a single cost object. An expenditure that can be directly traced in the organization’s management accounting system to a particular cost object, e.g. a pharmaceutical that is used exclusively for treating a particular diagnosis-related group (DRG) and no other [26]. 

The presence of charity wards in Philippine hospitals, whether public or private, has been a quiet issue. Often times the patients confined in these wards are the ones that provide the basic medical materials needed. There are also instances wherein professional fees of doctors are either waived or discounted for, while the hospital fee is rarely waived. The charity system of service was first practiced in St. Luke’s Medical Center and was established by American Missionaries in 1903. It has been cited that in UST, their charity wards have solely been funded via the paying wards and never through government subsidies nor through the tuition fees of students enrolled in medical and allied medical courses. It also shows that hospitals charge more than they really should but since it is for a good cause, it may well be worth it. It has also been general knowledge that in charity wards, medical interns and students are often allowed to handle patients while they have very limited patient in pay wards. It is something that hospitals will not publish but it is a widely accepted act. Also, faster and better service can obviously be found in pay wards and not in charity wards [31]. 

The GOLD 2007 classification (GOLD grades) was categorized as grade 1 (ppFEV1 ≥ 80), grade 2 (80 > ppFEV1 ≥ 50), grade 3 (50 > ppFEV1 ≥ 30), or grade 4 (ppFEV1 < 30). The GLI-2012 reference equation was used to calculate ppFEV1 and ppFVC [28]. The GOLD 2011 (ABCD groups) and GOLD 2017 classification (new ABCD groups) were categorized as group A, B, C, or D [29]. In the revised assessment scheme, patients should undergo spirometry to determine the severity of airflow limitation (i.e., spirometric grade). They should also undergo assessment of either dyspnea using mMRC or symptoms using CAT™. Finally, their history of moderate and severe exacerbations (including prior hospitalizations) should be recorded. The number provides information regarding severity of airflow limitation (spirometric grade 1 to 4) while the letter (groups A to D) provides information regarding symptom burden and risk of exacerbation which can be used to guide therapy [30]. 

At the basic level, a pack-year is defined as the equivalent of smoking one pack of cigarettes a day for one year. There are 20 cigarettes in a pack, so if a person smokes 20 cigarettes a day for one year, that’s literally one pack-year. However, there are all different gradations for this. If someone smokes two packs a day for a year, that’s two pack-years. Pack-years is a clinical and research approach that helps us better understand a person’s exposure to smoking [27]. 

## Methods

A prospective cross-sectional study was conducted for 6 months from the time of institutional ethics board approval by chart review. This study focused on out-of-pocket expenses of patients for hospitalization utilizing direct costing. A total enumeration of cases was employed for sample size. The subjects included in the study were admissions from both charity and pay wards. Private accommodations provide individual or shared rooms with air-condition units, and patients are primarily seen by specialist attending physicians. Further, the data extraction was done by the research coordinator who collects data daily. The cases are admitted under the internal medicine department and a census review is done to identify the COPD cases admitted.

### Procedure of the study

All admissions fulfilling the inclusion criteria within the pre-set period was included in the study. Baseline characteristics, diagnostic and therapeutic sheets review was done. Recognizing the variability in terms of costing for medications that was bought outside the institution, actual brand names used by each patient for their medicines has been recorded to allow for costing to be more accurately derived. For the diagnostic procedures, laboratory or imaging tests that were performed outside the institution were also recorded. Information was gathered directly from the nursing personnel/watchers caring for the patient. Direct costing for these medicines and procedures were inquired from patients/company or the actual establishments where these tests were bought or performed. Medications and diagnostic tests not indicated in the chart or not implemented or carried out were excluded as part of the direct costing. Other sources of direct costs such as syringes, IV lines, fluids, etc. were not included in this study due to difficulty in accounting its use. Chart reviews were done regularly at 1-3-day intervals during the course of stay, noting daily changes in the medications, or new diagnostic procedures ordered. Records from the billing section was consequently reviewed upon discharge to account for the professional fees. A data extraction form was used during chart review.

Data were encoded on Microsoft Word format for documentation and analysis was done in Microsoft Excel. Estimated total expenses were itemized as costs of accommodation, diagnostics, treatment, and professional fees as pre-specified. The total computed net expenditure incurred was compared to the actual case rate as follows:

*Total net expenditure* = Accommodation cost + Diagnostics cost + Therapeutics cost + Professional fess (if applicable).

*Out-of-pocket cost* = Total net expenditure – Phil-health case rate.

### Data Analysis

Descriptive statistics was used to summarize the demographic and clinical characteristics of the patients. Frequency and proportion were used for categorical variables, median and inter quartile range for non-normally distributed continuous variables, and mean and SD for normally distributed continuous variables. Independent Sample T-test, Mann-Whitney U test and Fisher’s Exact/Chi-square test were used to determine the difference of mean, rank and frequency, respectively, between charity and private patients. Odds ratio and corresponding 95% confidence intervals from binary logistic regression were computed to determine significant predictors for prolonged hospital stay and hospitalization cost of > $ 374.14. All statistical tests were two-tailed test. Shapiro-Wilk was used to test the normality of the continuous variables. Missing variables were neither replaced nor estimated. Null hypotheses were rejected at 0.05α-level of significance. STATA 13.1 was used for data analysis.

### Ethical consideration

Names of the research subjects were not indicated in the questionnaires and forms were safely stored in a secure location. The vulnerability of the elderly and socio-economically disadvantaged (charity) patients was considered in the study. The investigators ensured that the best level of care was given and social welfare assistance was provided irrespective of the patient’s decision to participate. Informed consent was obtained from all the subjects.There were no conflicts of interest identified among the investigators.

## Results

There were a total of 43 admissions between June 2019 to November 2019 at the Philippine General Hospital with a diagnosis of COPD exacerbation. Table 1 shows the basic demographic characteristics of the patients admitted. All of the patients included in this study had Philippine Health Insurance support, and zero private health maintenance organization (HMO) coverage. The mean age was significantly younger among patients admitted to charity service at 60 years old, while the mean age of patients admitted to private service was at 72 years old. Most of the admissions were predominantly males at 88%, with almost one half residing in a rural environment. Majority of the admissions between the two groups belonged to COPD Class D, and the most common comorbidities reported were hypertension, heart disease, diabetes, and cancer. The number of pack years were significantly higher among those patients admitted to private service at 47.5 years (mean) as compared to those under charity service at only 22.5 years (mean). The average length of stay was 8 days for both groups. There were no statistical difference between the two groups in terms of sex distribution, type of residence, GOLD classification, comorbidities, smoking history, and length of hospital stay.

**Table 1:** Demographic and clinical profile of the patients

	Total (n = 43)	Charity (n = 28, 65.12%)	Private (n = 15, 34.88%)	*P*-value
	Frequency (%); Mean ± SD; Median (IQR)			
Age 19 to 45 years old 45 to 64 years old > 65 years old	64.49 ± 12.68 3 (6.98) 20 (46.51) 20 (46.51)	60.43 ± 10.99 3 (10.71) 16 (57.14) 9 (32.14)	72.07 ± 12.47 0 4 (28.57) 10 (71.43)	**0.003** **0.042**
Sex				0.643
Male Female	38 (88.37) 5 (11.63)	24 (85.71) 4 (14.29)	14 (93.33) 1 (6.67)	
Type of Residence				1.000
Urban Rural	23 (53.49) 20 (46.51)	15 (53.57) 13 (46.43)	8 (53.33) 7 (46.67)	
Gold Classification				1.000
Class C Class D	10 (29.41) 24 (70.59)	7 (30.43) 16 (69.57)	3 (27.27) 8 (72.73)	
Comorbidities				
Hypertension Diabetes Heart disease Cancer	20 (46.51) 3 (6.98) 12 (27.91) 2 (4.65)	10 (35.71) 1 (3.57) 6 (21.43) 2 (7.14)	10 (66.67) 2 (13.33) 6 (40) 0	0.064 0.275 0.287 0.535
Number of pack years	30 (20 to 50)	22.5 (20 to 30)	47.5 (30 to 100)	**0.005**
Smoking history				0.073
Current Smoker Previous smoker Non-smoker	37 (86.05) 3 (6.98) 3 (6.98)	26 (92.86) 0 2 (7.14)	11 (73.33) 3 (20) 1 (6.67)	
Length of hospital stay	8 (6 to 12)	8.5 (7 to 11.5)	8 (6 to 31)	0.759

Table 2 shows the type of room accommodation among all COPD exacerbation admissions. The majority, or 51.16% were admitted to the charity non-ICU or regular ward/rooms, and 13.95% of these patients required ICU-level care. On the other hand, 32.56% and 2.33 were admitted ate private non-ICU and private ICU, respectively.

**Table 2:** Type of room of the patients (*n* = 43)

	Frequency (%)
Charity Non-ICU	22 (51.16)
Charity ICU	6 (13.95)
Private Non-ICU	14 (32.56)
Private ICU	1 (2.33)

Table 3 shows the hospitalization cost. In terms of price per day and total cost of hospitalization, it shows that there is a significant difference between private and charity services for it has a *p*-value of 0.00 which is lower than a 1% level of confidence. It unveils that the average price per day cost of hospital stay is significantly lower for the charity service at $ 75.89 compared to private service which is almost 4-fold at $ 285.71. Further, the total cost for hospitalization was significantly lower in the charity group compared to the private service patients, with the majority of the cost difference likely driven by the absence of professional fees in the former. On the other hand, medication cost, diagnostic tests cost, and procedural cost have no significant difference between private and charity service for it has *p* values of 0.575, 0.919, and 0.198, respectively which is greater than a 5% level of confidence.

**Table 3:** Hospitalization cost (peso-dollar rate of 56)

	Total (*n* = 43)	Charity (*n* = 28, 65.12%)	Private (*n* = 15, 34.88%)	*P*-value
	Median (IQR)			
Price per Day	107.13 (62.5 to 241 07)	75.89 (62.5 to 102.68)	285.71 (142.86 to 1049.11)	**< 0.001**
Professional fee	267.86 (142.86 to 303.57)	-	267.86 (142.86 to 303.57)	-
Medication cost	160 (122.50 to 209.11)	157.68 (102.64 to 186.07)	166.61 (118.75 to 438.57)	0.575
Diagnostic tests cost	148.75 (102.86 to 202.14)	147.86 (116.61 to 182.50)	148.75 (92.86 to 213.93)	0.919
Procedural cost	24.11 (5.71 to 72.86)	20.54 (5.54 to 55.54)	30.18 (10.18 to 90.54)	0.198
Total cost	503.27 (367.86 to 872 32)	408.39 (18.5 to 31.44)	916.43 (33.53 to 110.7)	**< 0.001**

**Fig. 1:**
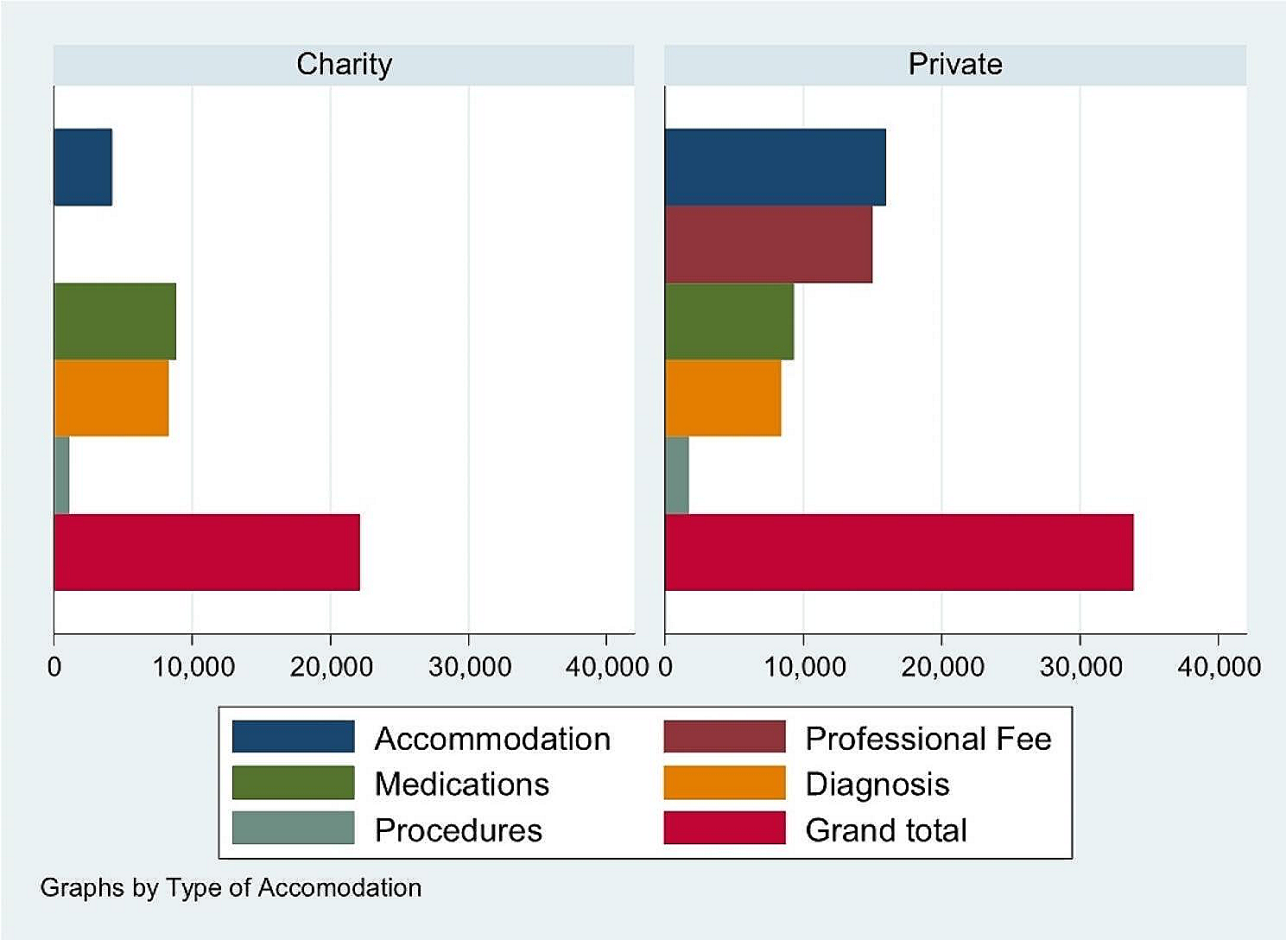
Median value of the hospitalization cost in Philippine pesos (Php) between Charity and Private patients

Figure 1 shows the groups admitted under charity service and private service and their breakdown of hospitalization costs itemized under accommodation, medications, procedures, professional fees, diagnosis/diagnostics, procedures, and grand total cost. The significant differences in cost between the two groups are in the price per day and total cost.

Table 4 shows the test for the significant predictors of prolonged hospital stay. It reveals that the profile of the respondents in terms of age, sex, type of residence, and GOLD classification does not significantly predict patients’ hospital stay for more than 9 days. Also, comorbidities and pack years of smoking are still not significant in influencing patients’ stays in the hospital. Moreover, the type of accommodation, whether private or public does not also, significantly predict patients stay in the hospital for > 9 days.

**Table 4:** Predictors of prolonged hospital stay

	Prolonged hospital stay		Odds ratio (95% CI)	*P*-value
	Yes (n = 18, 41.86%)	No (n = 25, 58.14%)		
	Frequency (%); Mean ± SD; Median (IQR)			
Age	66.39 ± 14.81	63.12 ± 11.04	1.02 (0.97‒	0.402
19 to 45 years old 45 to 64 years old > 65 years old	1 (5.56) 8 (44.44) 9 (50)	2 (8) 12 (48) 11 (44)	1.07) (reference) 1.33 (0.10‒17.3) 1.64 (0.13‒21.1)	- 0.826 0.706
Sex				
Male	14 (77.78)	24 (96)	(reference)	-
Female	4 (22.22)	1 (4)	6.86 (0.70‒67.6)	0.099
Type of Residence				
Urban Rural	10 (55.56) 8 (44.44)	13 (52) 12 (48)	(reference) 0.87 (0.26‒2.93)	- 0.818
Gold Classification				
Class A Class B Class C Class D	0 0 4 (40) 6 (60)	0 0 6 (25) 18 (75)	- - (reference) 0.5 (0.10‒2.40)	- - - 0.386
Comorbidities				
Hypertension Diabetes Heart disease Cancer	8 (44.44) 1 (5.56) 4 (22.22) 0	12 (48) 2 (8) 8 (32) 2 (8)	0.87 (0.26‒2.93) 0.67 (0.06‒8.09) 0.61 (0.15‒2.45) -	0.818 0.757 0.483 -
Number of pack years	30 (20 to 66)	30 (20 to 40)	1.01 (0.99‒1.03)	0.255
Smoking history				
Current Smoker Previous smoker Non-smoker	12 (66.67) 3 (16.67) 3 (16.67)	25 (100) 0 0	(reference) - -	- - -
Type of Accommodation				
Private Charity	6 (33.33) 12 (66.67)	9 (36) 16 (64)	0.89 (0.25‒3.18) (reference)	0.856 -

Table 5 shows that age, sex, type of residence, GOLD classification, comorbidities, pack years of smoking, and type of accommodation were not significant predictors of hospitalization cost of ≥ $ 357 for it obtained *p* values greater than 5% level of confidence.

**Table 5:** Predictors of hospitalization cost of ≥ $ 357

	Hospitalization cost of ≥ $ 357		Odds ratio (95% CI)	*P*-value
	Yes (n = 33, 76.74%)	No (n = 10, 23.26%)		
	Frequency (%); Mean ± SD; Median (IQR)			
Age 19 to 45 years old 45 to 64 years old >65 years old	63.3 ± 13.34 3 (9.09) 15 (45.45) 15 (45.45)	68.4 ± 9.83 0 5 (50) 5 (50)	0.97 (0.91‒1.03) - 1.0 (0.24‒4.18) (reference)	0.267 - 1.000 -
Sex				
Male Female	28 (84.95) 5 (15.15)	10 (100) 0	(reference) -	- -
Type of Residence				
Urban Rural	18 (54.55) 15 (45.45)	5 (50) 5 (50)	(reference) 0.83 (0.20‒3.44)	- 0.801
Gold Classification				
Class A	0	0	-	-
Class B Class C Class D	0 8 (30.77) 18 (69.23)	0 2 (25) 6 (75)	- (reference) 0.75 (0.12‒4.56)	- - 0.755
Comorbidities				
Hypertension Diabetes Heart disease Cancer	17 (51.52) 3 (9.09) 8 (24.24) 1 (3.03)	3 (30) 0 4 (40) 1 (10)	2.48 (0.54‒11.3) - 0.48 (0.11‒2.14) 0.28 (0.02‒4.95)	0.240 - 0.336 0.386
Number of pack years	30 (20 to 50)	30 (25 to 45)	1.002 (0.98‒1.0)	0.842
Smoking history				
Current Smoker Previous smoker Non-smoker	28 (84.85) 2 (6.06) 3 (9.09)	9 (90) 1 (10) 0	(reference) 0.64 (0.05‒7.95) -	- 0.731 -
Type of Accommodation				
Private Charity	14 (42.42) 19 (57.58)	1 (10) 9 (90)	6.63 (0.75‒58.6) (reference)	0.089 -

## Discussion

This study aimed to prospectively evaluate the overall cost of admissions for COPD exacerbation in a tertiary government hospital over a 6-month period. Despite the existence of Phil-health insurance, the provision of $ 435.71 as the case rate for this diagnosis is inadequate based on this study. With a total mean cost of $408.39 to $916.43 per hospitalization, out-of-pocket expenditure is unavoidable. The lowered total cost for hospitalization in the charity can be attributed to the majority of the cost difference likely driven by the absence of professional fees in the former. The medication, diagnostic tests, and procedural cost does not vary between private and charity hospitals. It can be inferred that patients’ profiles, health conditions, and quality of accommodation do not influence the hospitalization cost.

A study in the US on cost of COPD in terms of medical care and loss of productivity/absenteeism amounted to $36 billion, with costs projected to even increase for 2020 [20]. As much as 70% of cost of illness is attributed to COPD admissions [21]. A study by Pfuntner et al. in the US showed that COPD cost per stay was at $6,400 in 1997 which subsequently increased to $7,400 in 2010 with an accounted inflation of 15.6% after 13 years. COPD was among the 3 diagnoses with the highest aggregate cost in the US mainly due to its frequency [22].

There are variations to costs for different countries. Economic analysis on COPD from different countries reflected varied costs: $522 in France, $1258 in Canada, $3196 in Spain, and $4119 in USA, with the majority of these costs being derived from hospital admissions. Other main sources of direct cost include long term oxygen therapy and maintenance medications after discharge [23]. 

For our study, mean age was 64.49 + 12.68, with high prevalence of comorbid conditions. Pack years were not significant between patients admitted to the private service, and this could be attributed to the majority belonging in the older age group. The mean duration of hospital stay was 8 days. There were no significant predictors for cost and duration of stay identified in the present study, and this may be due to the small sample size. According to a study by Torabipour, 2 major drivers for cost for COPD were derived from hospitalizations and medications. Among hospitalized patients, major determinants of costs were history of hypertension, duration of hospital stay, and number of clinical consultations [13]. Therefore, limiting unnecessary clinical consultations, adequate management of comorbidities, and shortening hospitalization stay may potentially equate to lower hospitalization costs for COPD. Another and perhaps an important factor for added cost to patients is the short term and long term risk for re-admission. This study did not measure the following, and therefore represent potential limitations, including indirect costs and productivity losses, discharge medication costs and procedures (rehabilitation, long-term oxygen therapy).

## Conclusion

Our study indicates that the mean hospitalization cost for COPD exacerbation is $ 503.27 with significantly lower cost for charity admissions at $ 408.39 pesos vs. private admissions at $ 916.43. Despite existence of Phil-health, in-patient coverage for this disease is still insufficient. Majority of the patients were in the elderly age group, with a high prevalence of comorbidities. The present study was not able to identify major drivers for cost or prolonged hospitalization stay. However, measures such as maximizing COPD control in the out-patient setting, avoiding unnecessary tests/procedures, reducing hospital stay could potentially reduce total cost for this disease. We recommend similar studies that will employ a larger sample size, include indirect costs, and recruit patients from different institutions to account for variations in health care delivery systems and financial sources.

## Data Availability

Requests regarding raw data or other pertinent information concerning the study can be provided through electronic mail to the main investigator.
